# PsychArray-Based Genome Wide Association Study of Suicidal Deaths in India

**DOI:** 10.3390/brainsci13010136

**Published:** 2023-01-12

**Authors:** Chittaranjan Behera, Ruchika Kaushik, Deepak Ramkumar Bharti, Baibaswata Nayak, Daya Nand Bhardwaj, Dibyabhaba Pradhan, Harpreet Singh

**Affiliations:** 1 Department of Forensic Medicine & Toxicology, All India Institute of Medical Sciences, New Delhi 110029, India; 2 Department of Gastroenterology, All India Institute of Medical Sciences, New Delhi 110029, India; 3 Biomedical Informatics (BMI), ICMR, New Delhi 110029, India

**Keywords:** suicidal deaths, GWAS, PsychArray, single nucleotide polymorphisms (SNPs), genetic association, psychological autopsy

## Abstract

Background: Suicide is a preventable but escalating global health crisis. Genome-wide association studies (GWAS) studies to date have been limited, and some are underpowered. In this study, we aimed to perform the PsychArray-based GWAS study to identify single nucleotide variations associated with suicide in the Indian population. Methods: We recruited unrelated subjects who died by suicide as cases (N = 313) and the non-suicidal deaths as controls (N = 294). The 607 samples were genotyped, including cases and controls using the Illumina Infinium PsychArray-24 BeadChip v1.3 Results: In our study, four single nucleotide polymorphisms (SNPs) crossed the threshold of significance level <1 × 10^−5^. One of them is intronic at Chromosome2:rs1901851 and three are intergenic at Chromosome12:rs3847911, Chromosome8:rs2941489, Chromosome8:rs1464092. At a significance level of 5 × 10^−5^, we found a few more SNPs, with the majority of them being intergenic variants. The associated genes were associated with various important functions ranging from cell signaling, GTP binding, GPCR binding, and transcription factor binding. Conclusions: The SNPs identified in our study were not reported earlier. To our best knowledge, this study is one of the first GWAS for suicide in the Indian population. The results indicate few novel SNPs that may be associated with suicide and require further investigation. Their clinical significance is to be studied in the future.

## 1. Introduction

Suicide is the fourth most common cause of death for people between 15 to 19 years of ages, with >700,000 deaths each year worldwide [1]. The national suicide rate has risen to 12 per 100,000 population in the year 2021 from 11.3 in the year 2020 [2]. Suicide is most frequently associated with a psychiatric condition in 90% of cases. It is further linked to mood disorders in up to 60% of cases, but the majority of people who experience mood issues never attempt suicide [3]. There is a low willingness to seek psychiatric care in our nation because of the lack of awareness and stigma around mental health. In India, there is a 9% to 25% correlation between psychiatric illness and suicide, according to past studies [4].

There are genetic risks for suicidal behavior, evidenced by the research on twins [5], family-based studies [6], and adoption studies [7]. Until now, there have been no known markers or tools available to evaluate an individual’s risk of suicide. However, according to McGuffin et al. (2010), about 40% of all complex interactions of suicidal behavior can be attributed to the genetic components [8]. Mann et al. conceptualized a stress–diathesis model of suicidal behavior that considers a predisposition to the act (diathesis) and an acute precipitating factor (i.e., stress) [3,9,10]. Mood disorders, alcoholism, schizophrenia [11], and the diathesis for suicide [9,12] all have hereditary components. Finding the discrete and responsible gene for the diathesis has been difficult. Many studies have focused on the candidate genes approach related to the serotonin system, which has been more diligently associated with suicide. Ideally, we should look beyond the serotonin system to understand the complex interplays of suicide [13,14] and to prevent suicide [15].

Recently, several genome-wide association studies (GWAS) were carried out for identification of suicide risk variants. These GWAS studies are mostly population-based and identified variants, as suicide risk may differ in different ethnic populations. No such studies were carried for Indian populations. A recent GWAS study on suicide attempters targeting nearly 50 k subjects (with and without psychiatric disorders) isolated some loci, of which one, chromosome 2 [16], has been replicated successfully. Two were nominally replicated on chromosome 6 [17] and chromosome 3 [18], demonstrating that clinically predicted suicide shares considerable heritability [19]. Other GWAS studies for suicide risk were undertaken in conjunction with particular psychiatric diseases, which may raise concern over GWAS study reproducibility [20]. Another limitation is that the majority of suicide studies enrolled subjects had suicidal ideations and behavior, which are far more prevalent and common than completed suicide, and thus provide significant and sufficient power to the sampling. However, suicidal behavior can range from low-lethality, low-intent impulsive acts to high-lethality, high-intent suicidal acts. High lethality suicidal acts correlate more consistently with biological abnormalities in the brain [12]. Completed suicides represent the most lethal outcome of the act and should be considered a key phenotype for detecting a genetic association with a higher probability. Moreover, most of the GWAS studies in completed suicide subjects have been conducted in Caucasian populations, with only a handful in the Asian population, and none in the Indian population.

Genome-wide SNP (single nucleotide polymorphism) arrays have made possible a gene-centric, low-coverage, hypothesis-free survey of most genes [21,22,23], which implies that these studies will locate new or unanticipated candidate genes or regions in the genome closer to functionally important candidate genes with a pathogenic role [23]. PsychArray is an Illumina platform that provides coverage of all psychiatric disorder-related genes and many more. The content of PsychArray-24 includes 265,000 proven tag SNPs found on the Infinium Human Core-24 Bead Chip, 245,000 markers from the Infinium Exome-24 Bead Chip, and an additional 50,000 markers associated with common psychiatric disorders. Psychiatric disorders include schizophrenia, autism, bipolar disorder, ADHD, major depression, OCD, anorexia, and other disorders.

The objective of this study was to conduct India’s first PsychArray-based GWAS investigation to identify single nucleotide variants associated with suicide. We recruited the unrelated suicidal death subjects as cases and the non-suicidal deaths as controls. This research also aimed to study the psycho-social profile of the subjects with the help of psychological autopsies.

## 2. Materials and Methods

### 2.1. Subjects

The entire study was carried out at the Department of Forensic Medicine and Toxicology, collaborating with the Departments of Gastroenterology (Molecular) and Psychiatry at the All India Institute of Medical Sciences (AIIMS), New Delhi, India. As per institutional review board guidelines, the closest available family member’s (i.e., legally authorized relatives, LAR) written consent was obtained for sample collection and clinical data usage in the study. The semi-structured psychological autopsy proforma was created based on earlier published work by Ebert and Shneidman [24,25]. Details of the deceased were collected under 12 domains: (1) Socio-demographic details and family background, (2) history of suicidal behavior, (3) overall personality description, (4) perceived psychopathology 2 weeks prior to death, (5) self-care routine 2 weeks prior to death, (6) general coping behavior, (7) intrapersonal relationship, (8) medical history, (9) post-mortem findings, (10) history of genetic disorders, (11) history of drug use, and (12) reflective mental status. These details were obtained for each subject after interviewing one or more close family members or informants. All subjects were autopsied at AIIMS-New Delhi from 2017 to 2019. The samples were collected within 48 h of death with exclusion criteria, i.e., unknown/unidentified dead bodies, decomposed bodies, causes of death that were unclear, and deceased non-Indian descendants.

#### 2.1.1. Suicide Cases (n = 313)

Autopsies of suicide victims were performed at the Department of Forensic Medicine and Toxicology. The final verdict of the death reason (completed suicide) was made by the medical examiner and the government investigation agency (Police). Trained personnel interviewed LARs (family/informants) and reviewed medical records to gather background information on completed suicides using a semi-structured psychological autopsy protocol. Cases where there were very few details available about the deceased were excluded from the study.

#### 2.1.2. Non-Suicide Controls (n = 294)

Autopsies of non-suicide controls were also done in the same department. The non-suicide controls included death of the deceased by natural causes (myocardial infarction, CAD, etc.) or accidental (road traffic accidents, drowning, or electrocution) ensured by the post-mortem report. In addition to this, two other parameters were also considered to have non-biased controls: (i) A psychological autopsy was performed on the deceased, and (ii) medical records were reviewed to determine if any kind of psychiatric illness or suicidal behavior was present in the deceased; if such behavioural was discovered (for example, any mental illness, suicide attempts, or a desire to die), the literature suggests a strong association between psychiatric illness and suicidal behavior. Hence, at the time of recruitment of the subjects, individuals having psychiatric illness (n = 7) were excluded from the study and not genotyped.

#### 2.1.3. SAS Population from 1000 G Project

The South Asian population (SAS) from the 1000 Genomes Project was included as additional control samples for this study for population stratification and also for imputation purposes [26]. There are five sub-populations of the SAS population, including Sri Lankan Tamil from the UK (STU), Indian Telugu from the UK (ITU), Gujarati Indian from Houston, Texas (GIH), Punjabi from Lahore, Pakistan (PJL), and Bengali from Bangladesh (BEB).

### 2.2. Sample Collection and DNA Extraction

After taking informed consent from the LAR, 3 mL of femoral blood was collected in an EDTA vial and stored at −20 °C until extraction. DNA was extracted using the organic extraction method from 500 µL of whole blood. The quality and quantity of the DNA extracted were assessed using a Nano Drop ND-1000 spectrophotometer (Nano Drop Technologies, Wilmington, DE, USA). A DNA sample with a concentration of 5 ng/µL and an OD value (260/280 ratio) of around 1.8 is considered passable in terms of quality.

### 2.3. PsychArray Genotyping

The 607 samples (cases and controls) were genotyped using the Illumina Infinium PsychArray-24 BeadChip v1.3 (Illumina, San Diego, CA, USA) according to the manufacturer’s instructions. Genome Studio 2.0 was used to read the genotypes that were generated as .idat files.

### 2.4. Quality Control (QC)

**Figure 1:**
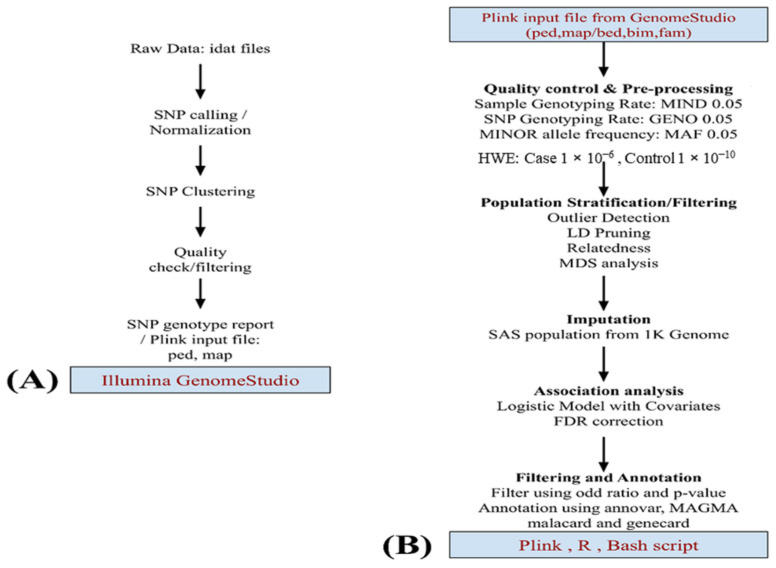
This flowchart depicts data analysis. (**A**) Starting with the raw image data file described in the methodology, pre-processing was performed using GenomeStudio v.2.0 and later using (**B**) PLINK v.1.9. Quality checking and preprocessing of data. The SAS population from 1 K genome phase 3 data was used for imputation. A logistic model was applied for the association studies using the MDS component and gender as covariates. Top markers in the study were annotated by Annovar, MAGMA, and more information was added using GeneCards and MalaCards.

The workflow for the analysis is illustrated in Figure 1. During genotyping analysis, 18 samples failed the array quality check and could not pass. The genotyped data (589 samples) were subjected to an extensive quality check using Genome Studio v2.0 [27,28] and converted into PLINK files for further processing. The second stage of QC procedures was performed using PLINK [29]. The SNPs were mapped with GRCh37 plus strand. Strand orientation has been corrected, and SNPs with >5% missing calls, and SNPs that failed the Hardy–Weinberg equilibrium (case: *p*-value < 1 × 10^−6^, control: *p*-value < 1 × 10^−10^) were excluded. A filter for samples with more than 5% missing calls was also applied. In both the case and control data sets, SNPs with minor allele frequency below 1% and those in build-specific LD regions were also removed from our study. After pre-processing, 574 (449 males and 125 females) samples remained for the analysis, which included 300 cases (212 males and 88 females) and 274 controls (237 males and 37 female). Similar QC steps were applied to the SAS population, which was later used for population stratification and additional control samples. Multidimensional Scaling Analysis (MDS) was performed using PLINK, including the SAS population, to identify individuals that deviate from the majority of sample structures.

### 2.5. Imputation

Imputation was carried out over the Michigan Imputation Server using the SAS population of 1000 Genomes phase 3 dataset [30]. The Michigan Imputation Server uses Eagle v2.4 for phasing and Minimac4 for imputation [31,32]. Hard call genotype output was filtered using the R2 score. SNPs with R2 < 0.3 were excluded, and the remaining genotypes were converted to PLINK format for further analysis.

### 2.6. Statistical Analysis and Functional Mapping

Demographic variables of the suicidal cases and non-suicidal controls were analyzed by a chi-square test for dichotomous variables and an independent *t*-test for continuous variables with the help of R software. Any value, i.e., a *p* value of 0.05 or lower, was considered significant.

**Figure 2:**
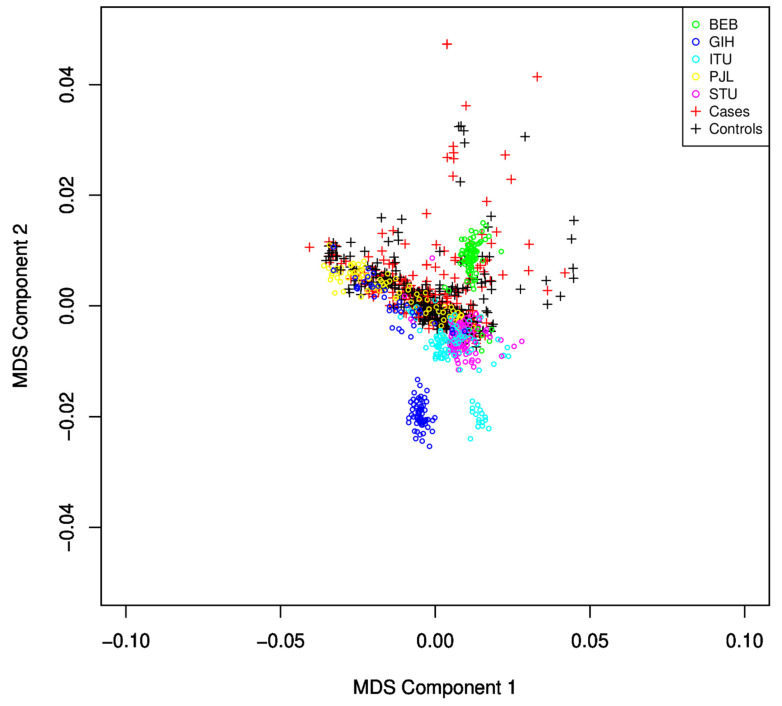
MDS plot for identification of population stratification and heterogeneity. The SAS population subgroup largely overlaps with the subjects under study. Samples with large deviations were discarded from the study.

GWAS Analysis: The remaining SNPs were converted into PLINK format for further processing after a quality check and pre-processing in Genome Studio. Additional QC and pre-processing were performed as recommended for the MIT imputation server for input files, and 447,072 SNPs passed the criteria and were ready for imputation. SNPs with an R2 score >= 0.3 were considered for filtering. Imputed dosage files converted back to hard call SNPs using DosageConvertor [33]. PLINK has been used for further quality checking as mentioned earlier, and similar steps were applied to the SAS population from the 1000 Genome dataset. The average genotyping call rate in the final dataset was 99.998%. The logistic regression model from PLINK was implemented to identify associations. The resulting association file was used for Manhattan plot and QQ-plot to identify potential associations and the quality of the analysis. A GWAS analysis has been carried out, including the SAS population. After identity by descent (IBD)analysis, the remaining SNPs were used for PLINK MDS analysis to identify and discard subjects that failed to follow population homogeneity. Those subjects were identified using component-wise outlier detection and by manual inspection of the MDS plot (Figure 2). The logistic regression model of PLINK has been applied using an additive genetic model adjusting for gender and the first ten MDS components. The GWAS logistic model SNP results were submitted to Annovar for functional annotation of SNPs [34], and gene set analysis was performed using MAGMA [35] with 50 Kb upstream and 10 Kb downstream regions. A total of 18,152 autosomal genes were available for analysis. For annotation and enrichment, GRCh37 build datasets were used in either tool. Gene-related data were obtained from GeneCard [36] and disease-related data were obtained from MalaCards and OMIM [37,38].

## 3. Results

### 3.1. Psycho-Social Profiles of the Subjects

The demographic attributes of both cases and controls are listed in Table 1. The majority of subjects, i.e., 81.46% of the suicidal group, fell into the age range of 11–40 years, while only 54.42% of the control group subjects fell into the same window. Males outnumbered females in both groups. There is a much larger significant difference (*p* < 1 × 10^−5^) in marital status between the two groups (59.74% married; 39.93% unmarried) in the suicidal cases than in the non-suicidal controls (76.87% married; 22.10% unmarried). Most of the cases were reported from the lower and lower-middle socio-economic strata of society. Hanging was the most common method of suicide, contributing 92.97%, followed by poisoning, falling from heights, and burning (single case). Only 5.75% of cases had a diagnosed psychiatric illness. Previous suicide attempts were present in 12.14% of the cases. Proportion tests were used to examine correlations between substance abuse and both groups. No significant relationship between substance abuse and the suicide or non-suicide group was observed (*p* = 0.463). Among the 313 suicidal deaths, subgroup analysis of suicidal behavioural predisposition was analyzed on the basis of previous attempt (12.14%), presence of a suicide note (4.15%), genetic inheritance (6.07%), psychological behavior prior to the commission of the act, and perceived or proven triggering factors.

### 3.2. Population Stratification/Multidimensional Scaling Analysis

We observed that, from the five sub-populations of SAS (Sri Lankan Tamil from the UK (STU) and Indian Telugu from the UK (ITU), Gujarati Indian from Houston, Texas (GIH), Punjabi from Lahore, Pakistan (PJL), and Bengali from Bangladesh (BEB)), many samples from GIH and ITU were found to be different from the population under study using the MDS plot (Figure 2) and were excluded from the study.

### 3.3. Inflation Rate of the Data/QQ Plot

**Figure 3:**
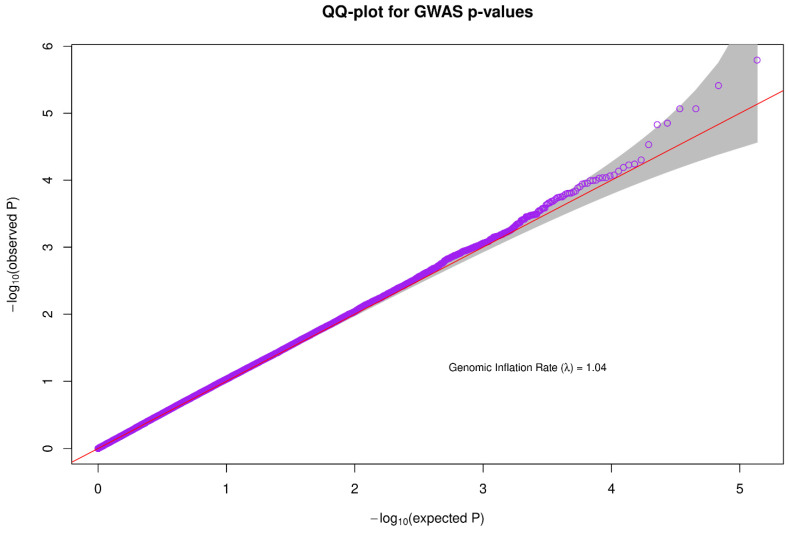
For association analysis, a quantile-quantile plot was used to evaluate the quality of the data. The genomic inflation rate was about 1.04, which is within an acceptable limit and is a reflection of high-quality data.

A Quantile-Quantile plot was used to check the quality of the data used for association analysis. The genomic inflation rate was approximately 1.04, which falls within the acceptable range and is an indicator of good quality data (Figure 3).

### 3.4. Association of SNPs with the Suicidal Deaths

**Figure 4:**
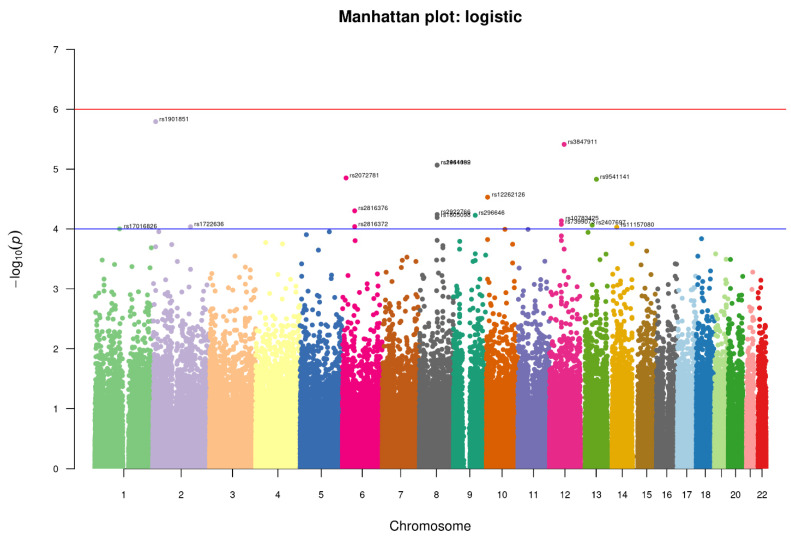
Manhattan-plot of *p*-values denoting top SNPs identified in the study. The two horizontal lines, blue and red, represents the GWAS significance threshold of −log_10_ *p* values i.e., <1 × 10^−4^ and <1 × 10^−6^ respectively.

We conducted an association analysis on 447,072 hard-called SNPs that passed quality control and were shared between controls and suicidal cases in this study. We used a relaxed *p*-value (1 × 10^−5^) for identifying any significant SNP (Figure 4). The top SNPs found with the relaxed *p*-value cut-off are enlisted in Table 2, which might be interesting for suicidal behavior.

Four SNPs crossed the threshold of significance level <1 × 10^−5^ spanning across the three chromosomes. One of them is intronic rs1901851 (*p*-value = 1.606 × 10^−6^, OR = 1.799), located on the chromosome 2 in close proximity to MIR3681HG, and three are intergenic, one being rs3847911 (*p*-value = 3.86 × 10^−6^, OR 1.814) on chromosome 12 located in the proximity of two genes, PPM1H and AVPR1, and the remaining two are located on chromosome 8 rs2941489 (*p*-value = 8.572 × 10^−6^, OR = 1.754) and rs1464092 (*p*-value = 8.572 × 10^−6^, OR = 1.754), whose nearest gene is HNFG. Additionally, at a significance level of 5 × 10^−5^, we found fourteen more SNPs, the majority of them being intergenic variants. The SNPs were functionally annotated using Annovar and MAGMA for their related genes. There were no SNPs found in the exome region. All SNPs were either intronic or intergenic. For most alleles, a relatively high odds ratio (range = 1.63–2.68) was observed (Table 2). As shown in Table 3, the associated genes of these SNPs have a variety of important functions, ranging from cell signaling to GTP binding, GPCR binding, and transcription factor binding.

The logistic regression model from PLINK was used to identify associations, and odds ratio after adjusting sex as a confounder.

## 4. Discussion

Our study is one of the PsychArray-based GWAS study in India in the context of suicide completers compared against non-suicide deaths. The objective of the study was to find out key variations that might be associated with a suicidal tendency. We selected subjects after a post-mortem and an extensive psychological evaluation of the deceased for the creation of a high-confidence dataset. In our study, we did not get many subjects with confirmed psychological disease. In the Indian population, due to the influence of cultural practices, psychological disorders do not gather much attention and are usually ignored; even close relatives of the deceased are unaware of the deceased’s mental health. However, the psychological autopsy proforma helped us identify subjects with strong signs of mental/psychological illness. Therefore, the share of diagnosed psychiatric illness among our study’s suicidal death group was found to be only in 5.75% of cases. To our best knowledge, this study is the first attempt to explore SNP-based heritability in completed suicide for any case-control GWAS study for Indian population exclusively. There was only one such study available in the Asian region (Japan) that tried to address suicidal heritability by exploring the GWAS for completed suicides (Otsuka et al., 2019). The area of completed suicide is largely unexplored, especially in India. Hence, our work is one of the first well-designed scientific efforts to identify genetic variations among suicidal deaths and non-suicidal deaths in India.

For high-quality data, extensive pre-processing was used, as recommended by the literature. SAS population has been incorporated in our study for imputation as well as additional control sets to strengthen our methodology and sample size. The samples are nearly moderate size due to strict selection criteria as, per power analysis, it is able to find strong associations. However, none of the SNPs in the study managed to reach the conventional significance level; each identified SNP requires further clinical study.

In our study, with the relaxed genome wide significance level <1 × 10^−5^, the top hit in our study, rs1901851 (Chr2; OR = 1.799; *p*-value = 1.606 × 10^−6^; MAF = 0.58), is in close proximity of the RNA gene MIR3681HH that may have regulatory function. The second SNP, i.e., rs3847911 on Chr12, was intergenic to the PPM1H and AVPR1A genes, whose molecular functions include phosphatase activity, RET signaling, and GPCR signaling. PPM1H is associated with neurological disorders such as ADHD [39] and endocrine diseases, while AVPR1A (Arginine Vasopressin) is a 7-transmembrane domain G-protein polypeptide that was reported to be involved in many neurological functions, including aggression, bonding, sex behavior, autism, and schizophrenia [40]. The third and fourth top SNPs (rs2941489, rs1464092) have a significance value <1 × 10^−5^. Both are associated with HNF4G and LINCO1111. HNF4G is associated with DNA binding, transcription factor binding, and steroid hormone receptor activity. It is associated with metabolic disorders such as onset of diabetes in the young or hyperuricemia and gastric or pancreatic cancers [41] (Table 1). LINCO1111 is associated with neurological diseases. rs1805098 is only exonic variant found in our study. It is also associated with the gene HNF4G, which has multiple roles, including carbohydrate metabolism, hormone receptor, and DNA-binding. Other related variants found in our study, which are related to this gene, are rs2922766 and rs1464092. A few SNPs with significance value < 1 × 10^−5^ were also found to be interesting. The rs2072781 variant found in the MYLIP gene’s UTR3 region is linked to the immune system. This gene is also called a post-transcriptional regulator of LDLR abundance. The *LXR-MYLIP-LDLR* pathway makes a complementary pathway available to sterol regulatory element-binding proteins for the feedback inhibition of cholesterol uptake [42]. Rs9541141 is located in close proximity to the RNA gene LINC00364, which may be regulatory in nature. Another important variation found is rs1722636 (CHR = 2, OR = 1.703; *p*-value = 9.19 × 10^−5^; MAF = 0.3567), whose nearest genes are RBMS1 and TANK. RBMS1 is associated with DNA binding and is associated with the blood disease ‘blue toe syndrome’, while TANK is associated with signal transduction and the TRAF pathway and is associated with infectious neurological disorders (Nipah virus encephalitis). One important variation is rs2816376, which is found near GCM1 and ELOVL5. GCM1 plays a role in DNA binding; the rs2816372 is another variant associated with this gene. ELOVL5 plays an important role in the elongation of fatty acid chains. Both are associated with cardiovascular blood disease, and rs296646 is associated with immune system signaling and is responsible for immunological disorders (Table 1).

As mentioned earlier, like many studies on attempted suicide cases, we obtained genes that are associated with various immunological, neurological, and infectious diseases. None of the identified SNPs, gene, or loci were earlier identified in previous GWAS studies. However, most of GWAS study has been conducted for the European and American ancestry and very limited attempts have been made for the Asian population, especially the Indian population. The major functional ontologies of identified genes, however—cell signaling, neurological effect, cell adhesion, and transcriptional activities—were discovered to overlap with previous studies.

## 5. Conclusions

Among the top candidate genes, we identified functional domains of transferase, kinases, immune responses, DNA binding, and signaling. Many top candidates are associated with CNS. We observed that MAF values in in-house controls are close to the SAS population, but there were cases (i.e., suicidal death group) where MAF values were relatively higher, as reflected by the odds ratio. The SNPs identified in our study were not previously reported. To the best of our knowledge, this study is one of the first GWAS conducted on suicidal death subjects in the Indian population. The results indicate few novel SNPs that may be associated with suicide and require further investigation.

## 6. Limitation

The moderate sample size of our GWAS study is the limitation of our study. As per conventional GWAS studies, we ended up with a smaller number of samples. Another limitation of the study is population stratification. The present SAS population does not entirely represent the Indian population. With a diversified ethnic Indian population, Delhi is a cosmopolitan city. The Indian population represents genetic diversity [43], and variable stratification levels complicate such studies, making them difficult to quantify. This is one of the study’s shortcomings.

## Figures and Tables

**Table 1:** Descriptive statistics for case and control’s socio-demographic data.

Characteristics	Cases Suicide Deaths (n = 313)	Controls Non-Suicidal Deaths (n = 294)	*p*-Value
Gender (Male)	220 (70.29%)	254 (86.39)	<1 × 10^−5 a^
Age (years (SD)	30.26 (11.58)	40.5 (14.48)	<1 × 10^−5 b^
Married Unmarried Separated	187 (59.74%) 125 (39.93%) 1 (0.31%)	226 (76.87%) 65 (22.10%) 3 (0.10%)	<1 × 10^−5 a^ <1 × 10^−5 a^ 0.346
Means of death Hanging Poisoning Burn Fall from height Natural Accidental Homicide	291 (92.97%) 17 (5.43%) 1 (0.31%) 4 (1.27%)	none none none none 216 (73.46%) 76 (25.85%) 2 (0.6%)	NA
Psychiatric illness	18 (5.75%)	none	NA
Family history of suicide	19 (6.07%)	none	NA
Previous suicide attempts	38 (12.14%)	none	NA
Suicide note	13 (4.15%)	NA	NA
Alcohol/Drug abuse	153 (48.88%)	134 (45.57%)	0.463 ^a^

^a^ Chi-Square Test, ^b^ Mann–Whitney test. The data depict the significant differences between gender and age in both groups.

**Table 2:** Genome-wide significant loci/SNPs from Psycharray.

S.No.	CHR	SNP	A1 (Minor)	A2	Cases Genotype	Controls Genotype	MAF Case	MAF Control	OR (95% CI)	*p*-Value	MAF 1 KG	MAF SAS
1	2	rs1901851	A	C	110/128/62	50/127/87	0.58	0.42	1.799	1.606 × 10^−6^	A = 0.5178 C = 0.4822	A = 0.518 C = 0.482
2	12	rs3847911	G	T	68/136/96	24/124/116	0.453	0.327	1.814	3.86 × 10^−6^	G = 0.2670 T = 0.7330	G = 0.403 T = 0.597
3	8	rs2941489	C	T	79/139/82	33/123/108	0.495	0.3579	1.754	8.572 × 10^−6^	C = 0.4127 T = 0.5873	C = 0.452 T = 0.548
4	8	rs1464092	C	T	79/139/82	33/123/108	0.495	0.3579	1.754	8.572 × 10^−6^	C = 0.4012 T = 0.5988	C = 0.453 T = 0.547
5	6	rs2072781	C	T	5/72/223	1/31/232	0.1367	0.0625	2.681	1.405 × 10^−5^	T = 0.8297 C = 0.1703	T = 0.876 C = 0.124
6	13	rs9541141	T	C	40/129/131	19/87/158	0.3483	0.2367	1.8	1.478 × 10^−5^	C = 0.7544 T = 0.2456	C = 0.706 T = 0.294
7	10	rs12262126	A	G	86/147/67	40/135/89	0.5317	0.4071	1.696	2.944 × 10^−5^	G = 0.4343 A = 0.5657	G = 0.472 A = 0.528
8	6	rs2816376	C	T	43/142/115	22/94/148	0.380	0.2613	1.71	4.979 × 10^−5^	T = 0.6296 C = 0.3704	T = 0.623 C = 0.377
9	8	rs2922766	T	C	72/156/72	36/136/92	0.5	0.3939	1.701	5.732 × 10^−5^	T = 0.3770 C = 0.6230	T = 0.473 C = 0.527
10	9	rs296646	C	T	58/134/108	22/113/129	0.4167	0.2973	1.683	5.915 × 10^−5^	T = 0.6100 C = 0.3900	T = 0.598 C = 0.402
11	8	rs1805098	G	A	89/143/68	89/115/60	0.535	0.6079	1.663	6.447 × 10^−5^	G = 0.3852 A = 0.6148	G = 0.496 A = 0.504
12	12	rs10783425	C	T	65/147/88	29/126/109	0.4617	0.3484	1.661	7.325 × 10^−5^	C = 0.4998 T = 0.5002	C = 0.394 T = 0.606
13	12	rs7399073	C	A	43/144/113	17/111/136	0.3833	0.2746	1.709	8.376 × 10^−5^	C = 0.3844 A = 0.6156	C = 0.307 A = 0.693
14	13	rs2407697	G	T	17/133/150	10/73/181	0.2783	0.1761	1.838	8.627 × 10^−5^	G = 0.1683 T = 0.8317	G = 0.225 T = 0.775
15	6	rs2816372	G	A	60/145/95	32/106/126	0.4417	0.3219	1.634	9.14 × 10^−5^	A = 0.6148 G = 0.3852	A = 0.578 G = 0.422
16	2	rs1722636	T	C	40/134/126	14/104/146	0.3567	0.25	1.703	9.19 × 10^−5^	C = 0.7113 T = 0.2887	C = 0.691 T = 0.309
17	14	rs11157080	T	C	67/168/65	42/125/97	0.5033	0.3958	1.655	9.338 × 10^−5^	C = 0.6410 T = 0.3590	C = 0.540 T = 0.460
18	1	rs17016826	C	A	6/78/216	2/39/223	0.15	0.0814	2.242	9.984 × 10^−5^	A = 0.8986 C = 0.1014	A = 0.860 C = 0.140

**Table 3:** Nearest genes of the associated SNPs, their functions, and their association with the diseases.

S.No.	CHR	SNP	Function of Reference Gene	Nearest Gene (s)	Function	Diseases Associated with Gene (s)
1	2	rs1901851	Intron Variant	MIR3681HG	RNA Gene, and is affiliated with the lncRNA class.	
2	12	rs3847911	Intergenic	PPM1H;AVPR1A	PPM1H (Protein Phosphatase, Mg^2+^/Mn^2+^ Dependent 1H) is a Protein Coding gene. The protein encoded by AVPR1A gene acts as receptor for arginine vasopressin	PPM1H: Diseases associated with PPM1H include Multiple Endocrine Neoplasia, Type Iv and Attention Deficit-Hyperactivity Disorder. Gene Ontology (GO) annotations related to this gene include phosphoprotein phosphatase activity. An important paralog of this gene is PPM1J; AVPR1A: Diseases associated with AVPR1A include Acth-Independent Macronodular Adrenal Hyperplasia and Diabetes Insipidus. Among its related pathways are RET signaling and Signaling by GPCR.
3	8	rs2941489	intergenic	HNF4G;LINC01111	HNF4G (Hepatocyte Nuclear Factor 4 Gamma) is a Protein Coding gene; LINC01111 (Long Intergenic Non-Protein Coding RNA 1111) is an RNA Gene, and is affiliated with the lncRNA class	Diseases associated with HNF4G include Maturity-Onset Diabetes Of The Young and Hyperuricemia. Among its related pathways are regulation of beta-cell development and Gene Expression. Gene Ontology (GO) annotations related to this gene include DNA-binding transcription factor activity and steroid hormone receptor activity. An important paralog of this gene is HNF4A. Diseases associated with LINC01111 include Chromosome 8Q21.11 Deletion Syndrome and Sclerocornea.
4	8	rs1464092	intergenic	HNF4G;LINC01111		
5	8	rs2922766	intergenic	HNF4G;LINC01111		
6	6	rs2072781	UTR3	MYLIP	MYLIP protein interacts with myosin regulatory light chain and inhibits neurite outgrowth.	Diseases associated with MYLIP include Deafness, Autosomal Dominant 31, and Deafness, Autosomal Dominant 21. Among its related pathways are Lipoprotein metabolism and Innate Immune System.
7	13	rs9541141	intergenic	LINC00364	LINC00364 (Long Intergenic Non-Protein Coding RNA 364) is an RNA Gene and is affiliated with the lncRNA class.	-
8	10	rs12262126	intergenic	CALML3;LINC02657	CALML3 may function as a specific light chain of unconventional myosin-10 (MYO10), also enhances MYO10 translation, possibly by acting as a chaperone for the emerging MYO10 heavy chain protein. LINC02657 (LASTR) is an RNA Gene, and is affiliated with the lncRNA class.	Diseases associated with CALML3 include Alzheimer’s disease. Among its related pathways are tuberculosis and Inositol phosphate metabolism (KEGG). Gene Ontology (GO) annotations related to this gene include calcium ion binding.
9	6	rs2816376	Intergenic	GCM1;ELOVL5	GCM1 encodes a DNA-binding protein with a gcm-motif (glial cell missing motif) ELOVL5. It is highly expressed in the adrenal gland and testis and encodes a multi-pass membrane protein that is localized in the endoplasmic reticulum. This protein is involved in the elongation of long-chain polyunsaturated fatty acids.	Diseases associated with GCM1 include Cardiomyopathy, Familial Restrictive, 2 and Pre-Eclampsia. Among its related pathways are Human Early Embryo Development and Parathyroid hormone synthesis, secretion, and action. Diseases associated with ELOVL5 include Spinocerebellar Ataxia 38 and Intermittent Squint. Among its related pathways are alpha-linolenic (omega3) and linoleic (omega6) acid metabolism and Metabolism.
10	6	rs2816372	Intergenic	GCM1;ELOVL5		
11	9	rs296646	intergenic	SYK;LOC100129316	SYK gene encodes a member of the family of non-receptor type Tyr protein kinases. This protein is widely expressed in hematopoietic cells and is involved in coupling activated immunoreceptors to downstream signaling events that mediate diverse cellular responses, including proliferation, differentiation, and phagocytosis. LOC100129316 is a disease associated with CALML3, including Alzheimer’s Disease. Among its related pathways are Tuberculosis and Inositol phosphate metabolism (KEGG). Gene Ontology (GO) annotations related to this gene include calcium ion binding RNA Gene and is affiliated with the lncRNA class.	Diseases associated with SYK include Peripheral T-Cell Lymphoma and Hantavirus Pulmonary Syndrome. Among its related pathways are B cell receptor signaling pathway (KEGG) and signaling by GPCR.
12	8	rs1805098	Exonic (mis sense)	HNF4G	HNF4G (Hepatocyte Nuclear Factor 4 Gamma) is a Protein Coding gene	Diseases associated with HNF4G include Maturity-Onset Diabetes Of The Young and Hyperuricemia. Among its related pathways are regulation of beta-cell development and Gene Expression. Gene Ontology (GO) annotations related to this gene include DNA-binding transcription factor activity and steroid hormone receptor activity.
13	12	rs10783425	intergenic	POU6F1;DAZAP2	POU6F1: DNA-binding transcription factor activity. An important paralog of this gene is POU6F2. DAZAP2. This gene encodes a proline-rich protein which interacts with the deleted in azoospermia (DAZ) and the deleted in azoospermia-like gene through the DAZ-like repeats. This protein also interacts with the transforming growth factor-beta signaling molecule SARA (Smad anchor for receptor activation), eukaryotic initiation factor 4G, and an E3 ubiquitinase that regulates its stability in splicing factor containing nuclear speckles. The encoded protein may function in various biological and pathological processes, including spermatogenesis, cell signaling and transcription regulation, formation of stress granules during translation arrest, RNA splicing, and pathogenesis of multiple myeloma. Multiple transcript variants encoding different isoforms have been found for this gene.	POU6F1: Diseases associated with POU6F1 include Clear Cell Adenocarcinoma Of The Ovary and Clear Cell Adenocarcinoma. DAZAP2: Diseases associated with DAZAP2 include Thyroid Hormone Resistance, Selective Pituitary, and Azoospermia. Among its related pathways are Diurnally Regulated Genes with Circadian Orthologs. Gene Ontology (GO) annotations related to this gene include WW domain binding
14	12	rs7399073	Intergenic (2KB Upstream Variant)	POU6F1;DAZAP2		
15	13	rs2407697	intronic	RCBTB1	In rats, over-expression of this gene in vascular smooth muscle cells induced cellular hypertrophy. In rats, the C-terminus of RCBTB1 interacts with the angiotensin II receptor-1A	Diseases associated with RCBTB1 include Retinal Dystrophy With Or Without Extraocular Anomalies and Reticular Dystrophy Of Retinal Pigment Epithelium. An important paralog of this gene is RCBTB2.
16	2	rs1722636	intergenic	RBMS1;TANK	RBMS1 (RNA Binding Motif Single Stranded Interacting Protein 1) is a Protein Coding gene encodes a member of a small family of proteins which bind single stranded DNA/RNA. The TRAF (tumor necrosis factor receptor-associated factor) family of proteins associate with and transduce signals from members of the tumor necrosis factor receptor superfamily. The protein encoded by this gene is found in the cytoplasm and can bind to TRAF1, TRAF2, or TRAF3, thereby inhibiting TRAF function by sequestering the TRAFs in a latent state in the cytoplasm.	Diseases associated with RBMS1 include Blue Toe Syndrome and Diffuse Glomerulonephritis. Gene Ontology (GO) annotations related to this gene include nucleic acid binding and RNA binding. An important paralog of this gene is RBMS3. Diseases associated with TANK include Nipah Virus Encephalitis. Among its related pathways are Activated TLR4 signaling and TRAF Pathway. Gene Ontology (GO) annotations related to this gene include ubiquitin protein ligase binding.
17	14	rs11157080	intergenic	FBXO33;LINC02315	FBXO33 may be associated with placental RNAse inhibitor, and locus may be associated with copy number variation of UGT2B17 (GeneID 7367), which has been associated with susceptibility to osteoporosis (bone disease). LINC02315: Long Intergenic Non-Protein Coding	Diseases associated with FBXO33 include Protoplasmic Astrocytoma and Attention Deficit-Hyperactivity Disorder
18	1	rs17016826	Intergenic	LINC01677;LINC01661	LncRNA	-

## Data Availability

The data that support the findings of this study are available on request from the corresponding author [C.B.]. The data are not publicly available due to due to confidential restrictions applicable to health data but could be available under request and prior consent of the Institute’s Ethics Committee.
